# Effect of Astragalus Polysaccharides on Cardiac Dysfunction in db/db Mice with Respect to Oxidant Stress

**DOI:** 10.1155/2018/8359013

**Published:** 2018-11-15

**Authors:** Wei Chen, Qilin Sun, Jing Ju, Wenjie Chen, Xuelan Zhao, Yu Zhang, Yehong Yang

**Affiliations:** ^1^Department of Geriatrics, Huashan Hospital, Fudan University, Shanghai 200040, China; ^2^Department of Endocrinology, Huashan Hospital, Fudan University, Shanghai 200040, China

## Abstract

**Objective:**

Oxidant stress plays an important role in the development of diabetic cardiomyopathy. Previously we reported that Astragalus polysaccharides (APS) rescued heart dysfunction and cardinal pathological abnormalities in diabetic mice. In the current study, we determined whether the effect of APS on diabetic cardiomyopathy was associated with its impact on oxidant stress.

**Methods:**

Db/db diabetic mice were employed and administered with APS. The hematodynamics, cardiac ultra-structure, apoptosis, and ROS formation of myocardium were assessed. The cardiac protein expression of apoptosis target genes (Bax, Bcl-2, and caspase-3) and oxidation target genes (Gpx, SOD2, t/p-JNK, catalase, t/p-p38 MAPK, and t/p-ERK) were evaluated, respectively.

**Results:**

APS therapy improved hematodynamics and cardinal ultra-structure with reduced apoptosis and ROS formation in db/db hearts. In addition, APS therapy inhibited the protein expression of apoptosis target genes (Bax, Bcl-2, and caspase-3) and regulated the protein expression of oxidation target genes (enhancing Gpx, SOD2, and catalase, while reducing t/p-JNK, t/p-ERK, and t/p-p38 MAPK) in db/db hearts.

**Conclusion:**

Our findings suggest that APS has benefits in diabetic cardiomyopathy, which may be partly associated with its impact on cardiac oxidant stress.

## 1. Introduction

Diabetes mellitus (DM) is a metabolic disorder with multiple etiology and is one of the three major chronic diseases that affect human health. The morbidity and prevalence of DM is increasing at an alarming rate around the world including China [1]. At present, the prevalence of adults with diabetes in our country has reached 9.7 %, similar to the prevalence in European and American countries [2]. DM caused by chronic hyperglycemia is associated with both cardiovascular and microvascular complications which is the main cause of disability or death in diabetes [3]. Among them, especially the damage of the diabetic cardiomyopathy hazard is serious [4]. A condition termed as diabetic cardiomyopathy (DCM) is common in diabetic individuals independent of hypertension and coronary artery disease [5–7]. The characteristic cardiomyopathy of ventricular hypertrophy and cardiac dysfunction which can be detected by cardiac tissue Doppler at present as a unique diabetic complication is well recognized [8]. Although the underlying cause is multifactorial, diabetic cardiomyopathy has now been further emphasized. It is not only a significant cause of morbidity and mortality of cardiovascular complications in diabetic patients worldwide, but also a leading cause of end-stage heart failure [9].

The pathophysiology of diabetic cardiomyopathy is probably multifactorial. However, several studies have shown that sustained hyperglycemia is an independent risk factor which induces cardinal cells of redox imbalance [10]. Thus hyperglycemia-induced redox imbalance has become one of the important pathogenesis directly associated with diabetic cardiomyopathy [11]. High blood glucose-induced mitochondria ROS overproduction and myocardial cell oxidant stress damage criterion have been considered as the main driving force for the development of diabetes complications, both microvascular and cardiovascular [12]. Mitochondria play a pivotal role in energy generation, intermediary metabolism, and cell death, and they are primary sources of reactive oxygen species (ROS) such as superoxide within the cell and the major target for their damaging effects [13, 14]. The metabolic abnormalities (such as exposed to high glucose levels, increased oxidation of fatty acids and abnormal activation of angiotensin system) of diabetes cause mitochondrial overproduction of the reactive oxygen species (ROS) in endothelial cells of both large and small vessels and also in the myocardium. The excessive ROS in the heart and vascular stemming from several enzymatic sources is the central and major mediator of diabetes tissue damage, which is induced by excessive activation of myocardial apoptosis signaling pathways. The phenomenon of mitochondrial ROS overproduction leads to a vicious cycle of enhance oxidant stress and mitochondrial dysregulation, triggers the irreversible myocardial cell damage, and eventually leads to an increased risk for diabetic myocardial tissue remodeling and cardiac failure, namely, the diabetic cardiomyopathy [15].

Astragalus polysaccharide (APS) is one of the main active ingredients of Astragalus membranaceus, a traditional herbal Chinese medicine. The polysaccharides are a mixture of APS I and II. APS I is a kind of heterosaccharide which is composed of D-glucose, D-galactose, and L-arabinose in molar ratios of 1.75:1.63:1, with the average molecular weight of 36.3 kDa. APS II is a kind of dextran with high molecular weight, bonded mainly with a-(1→4)-D-glycosidic linkages [16]. Our previous study has demonstrated that APS treatment (at the dosage of either 1.0g/kg body weight per day or 2.0g/kg body weight per day) was sufficient to improve the systemic metabolic disorder and cardiac dysfunction in diabetic mice, and there was also no significant difference between mice with different APS dosage in the effect of APS on diabetic mice [17]. In addition, APS can significantly rescue the cardiac dysfunction and the abnormal hematodynamics in several kinds of diabetic model mice with cardiomyopathy, including STZ-induced diabetic mice with type 1 diabetes [17], NOD mice with type 1 diabetes DM [18], db/db mice with type 2 diabetes [19], and MHC-PPAR *α* mice with myocardial lipotoxicity [20].

Previously, we also found that APS could enhance the superoxide dismutase (SOD) activities and cardiac H_2_O_2_ formation in both STZ-induced mice with type 1 diabetes and SOD2+/- mice [21]. To our knowledge, the underlying pathogenesis and underlying mechanisms of the DCM are not completely understood. Whether APS therapy exerted direct effects which was independent of its influence on hyperglycemia or hyperlipidemia on diabetic-induced oxidant stress in the diabetic cardiomyopathy has not previously been determined.

Our research aims to further explore the potential mechanism and the correlation between oxidant stress, cardiomyopathy, and APS therapy. Therefore, in our current study, diabetic db/db mice (models of type 2 diabetes) were characterized and administrated with or without APS treatment, and the cardiac apoptosis and oxidation were evaluated accordingly, to investigate whether APS may improve diabetic cardiomyopathy through the inhibition of oxidant stress-mediated apoptosis in diabetic hearts.

## 2. Materials and Methods

### 2.1. Animals Experiments

Male homozygous db/db mice (B6.Cg-m+/+ Leprdb/J in C57BL/6J background) were obtained from, Jackson Laboratories, Bar Harbor, USA. Five-week-old db/db mice were administrated either APS (2000 mg/kg body weight per day) or normal saline by lavage for 15 weeks. APS were obtained from Shanghai Institute of Physiology Academia Sinica, China [16]. Age-matched male C57BL/6J mice (Laboratory Animal Unit, Fudan University, School of Medicine, China) were taken as the normal control. Optimal in vivo concentration of APS was decided on the basis of our earlier studies [20]. At the end of experiment, all mice were survived and sacrificed by cervical dislocation at 20 weeks of age, and the hearts were harvested for further experiments. Body weight was tested at 5 weeks and 20 weeks of age (Table 1).

The current study protocol was approved by the Institutional Animal Care and Use Committee of Shanghai Fudan University Medical Center and conformed to the guidelines of the National Research Council for laboratory animal care in research.

### 2.2. Left Ventricular Hematodynamics

Before sacrifice, mice were anesthetized with ketamine chloride at 40 mg/kg of body weight by intraperitoneal injection (*i.p.*; Imalgene, Merial Milano, Italy) plus medetomidine hydrochloride at 0.15 mg/kg of body weight (*i.p.*; Domitor, Pfizer Italia S.r.l., Latina, Italy). Invasive hematodynamic data were then recorded respectively. A microtip pressure transducer catheter (Millar SPC-320, Millar Instruments, USA) was advanced into the left ventricle to measure the left ventricular end diastolic diameter (LVEDD), the left ventricular end-systolic diameter (LVESD), and the left ventricular ejection fraction (LVEF). Echocardiographic images were obtained with the Acuson Sequoia 256 Echocardiography System (Acuson, Mountain View, USA) with methods for measurements and chamber size using M-mode, as previously reported [21].

### 2.3. Transmission Electron Microscopy Analysis

Left ventricular tissues were harvested after sacrifice, and were evaluated by transmission electron microscopy to define ultra-structural pathological changes. Cardinal samples were fixed in diluted Karnovsky's fixative and processed. Sections were cut on a Leica UCT ultra microtome at 80nm with a diamond knife, and stained with uranyl acetate and lead citrate. Images were viewed and photographed on a Philips Morgagni electron microscope (Philips, Amsterdam, the Netherlands), as previously reported [21].

### 2.4. Immunohistochemistry

After sacrifice, left ventricular tissues were harvested, fixed by formalin, and embedded in paraffin. 4 *μ*m sections were cut. Sections of myocardium were stained with the In Situ Cell Death Dection Kit, AP (Roche Diagnostics, Indianapolis, IN, USA) for the terminal deoxynucleotidyl transference-mediated dUTP nick-end labeling (TUNEL) assay as described in the manufacture's instructions. Replicated sections were analyzed to estimate the percentage of apoptotic cardiomyocytes by TUNEL assay accordingly.

### 2.5. ROS Detection in Myocardium

Reactive oxygen species (ROS) production in myocardium was measured by using the fluorescent dye 5-(6)-chloromethyl-2′,7′-dichlorodihydrofluorescein diacetate (CM-H_2_DCFDA, Invitrogen, Molecular Probes, USA). Briefly, myocardium were loaded with CM-H_2_DCFDA for 30 minutes. The signal generated by CM-H_2_DCFDA was directly proportional to myocardial H_2_O_2_ and ·OH concentration. Nuclei were stained by Syto17 capable of entering living cells and binding to the DNA. The generation of fluorescence calibration curves and the evaluation of cell brightness were measured using InSpeck Microscopy Image Intensity Calibration microspheres (Invitrogen, Molecular Probes, USA), and ROS formation were measured using Bio-Rad Radiance 2100MP multiphoton microscope and ImagePro analysis software.

### 2.6. Western Blotting

Left ventricular protein content was analyzed using Western blots (n=6 samples per group). Briefly, tissues were washed with phosphate-buffed saline and then suspended in 100*μ*L of the lysis buffer (150 mM NaCl, 20 mM Tris-HCL, 1% Triton X-100, 1mM EDTA, protease inhibitor cocktail, 10 mM NaF, and 2 mM Na3VO4, pH 7.5). The amount of proteins was valued by the Bio-Rad protein assay (Bio-Rad, Hercules, CA) and then loaded in each lane with equal amount. Separated by SDS-PAGE (12% SDS-poly-acrylamide gel electrophoresis), the proteins were transferred electrically to a polyvinylidene difluoride membrane. After blocking the membrane with 5% skim milk, all proteins were immunodetected using the rabbit anti-mouse different targeted antibodies. Primary antibodies against Bax, Bcl-2, caspase-3, Glutathione Peroxidase (Gpx), SOD2, total c-Jun N-terminal kinase (t-JNK), phosphorylated JNK (p-JNK), and catalase were purchased from Transduction Laboratories (USA), Santa Cruz Inc (CA), Abcam Inc. (USA), Upstate (USA), and Sigma (USA), respectively. The antibodies against phosphorylated p38 MAP kinase (p-p38 MAPK), total p38 MAP kinase (t-p38 MAPK), total extracellular signal-regulated kinase (t-ERK), phosphorylated extracellular signal-regulated kinase (p-ERK), and GAPDH were purchased from Cell Signaling, USA. GAPDH was detected as loading control. The horseradish peroxidase-conjugated anti-rabbit IgG was applied as the secondary antibody. All protein bands corresponding to targeted antibodies were scanned for quantification of protein expression levels. The densities of the bands in samples of the db/db mice with or without APS treatment were normalized to the densities of the corresponding bands in the C57BL/6J normal control.

### 2.7. Statistical Analysis

Results are expressed as means ± standard error of the mean (SEM). Comparisons of data between the two groups were made using unpaired Student's* t*-tests with GraphPad Prism 5 (GraphPad, San Diego, CA, USA). A* P*-value <0.05 was considered statistically significant.

## 3. Results

### 3.1. Effects of APS on Ventricular Hematodynamics in db/db Mice

**Figure 1:**
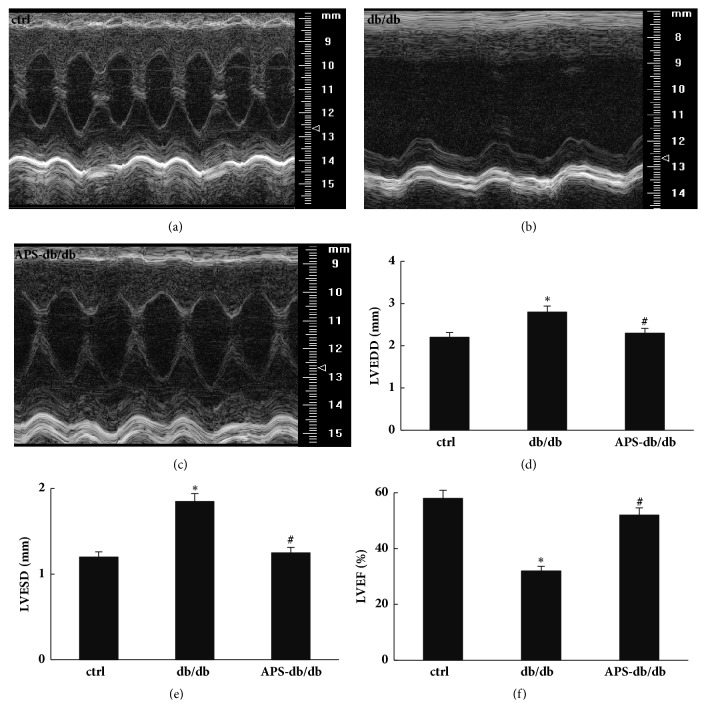
**Effects of APS on left ventricular hematodynamics in db/db mice.** After anesthetization, two-dimensional targeted M-mode echocardiography images were taken, and left ventricular hemodynamic parameters were measured in C57BJ/6J mice and db/db mice with or without APS treatment (n=6 per group). (a)~(c) Representative M-mode echocardiographic images. (d)~(f) Left ventricular hematodynamic parameters. LVEDD: left ventricular end diastolic diameter; LVESD: left ventricular end-systolic diameter; LVEF: left ventricular ejection fraction. Values were presented as mean ± standard error of the mean (SEM). *∗*P<0.05 versus C57BJ/6J control mice, and #P<0.05 versus db/db mice.

Echocardiography and hematodynamics were measured to evaluate cardiac function. In comparison with normal control mice, db/db mice exhibited significantly marked signs of systolic and diastolic ventricular dysfunction, including a significant reduction in LVEF, associated with a dilation of both LVEDD and LVESD (Figure 1). Notably, APS treatment significantly abolished the negative impact of diabetes on left ventricular hematodynamics and all parameters (including LVEDD, LVESD, and LVEF) reached the normal control values (Figure 1). This may suggest the prevention of APS on left ventricular dysfunction in diabetic mice, which was in line with our previous reports about the protection of APS on STZ-induced diabetic heart [21].

### 3.2. Effects of APS on Cardiac Ultrastructural Pathological Changes in db/db Mice

**Figure 2:**
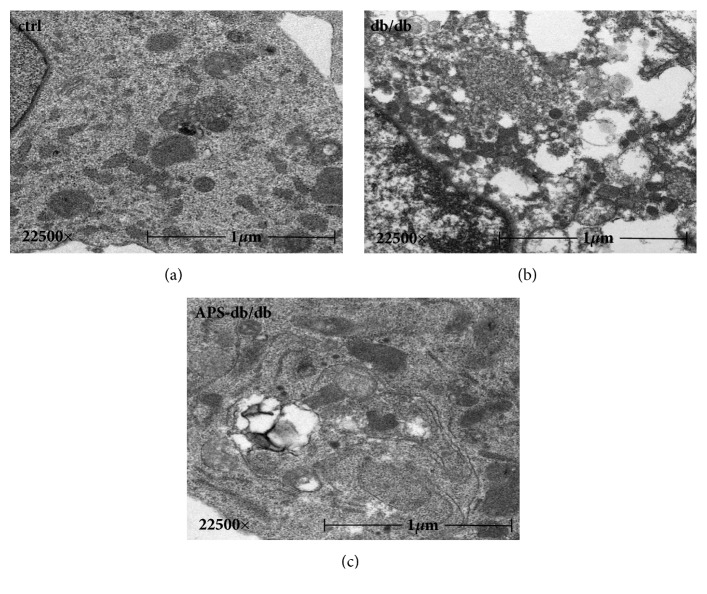
**Effects of APS on ultra-structural pathological changes of myocardium in db/db mice.** Left ventricular samples from C57BJ/6J mice and db/db mice with or without APS treatment (n=6 per group) were prepared and evaluated by transmission electron microscopy (TEM) analysis. (a)~(c) Representative transmission electron microscopy photographs of cardiomyocytes. Disrupted tubes, sarcomeres, and mitochondrial cristae were noted in cardiac myocytes from db/db mice (b), whereas TEM Features in hearts from APS-treated db/db mice (c) showed nearly normal-appearing mitochondrial cristae, tubes, and sarcomeres that resembled those from normal control (a) (original magnification:×22500).

Left ventricular samples were evaluated for the ultra-structural pathological changes by transmission electron microscopy (TEM) analysis (Figure 2). Hearts from db/db mice exhibited severe damage, including disruption of mitochondrial cristae, tubes, and sarcomeres (Figure 2(b)). In comparison, the ultra-structural pathological changes in cardiomyocytes from APS-treated db/db mice were unremarkable and resembled those from the normal controls (Figure 2(c)), which illustrated the protective effects of APS on myocardial ultra-structure in diabetic mice. This finding was exact in line with our previous reports about the protection of APS on STZ-induced diabetic heart [21].

### 3.3. Effects of APS on Diabetes-Induced Cardinal Apoptosis in db/db Mice

**Figure 3:**
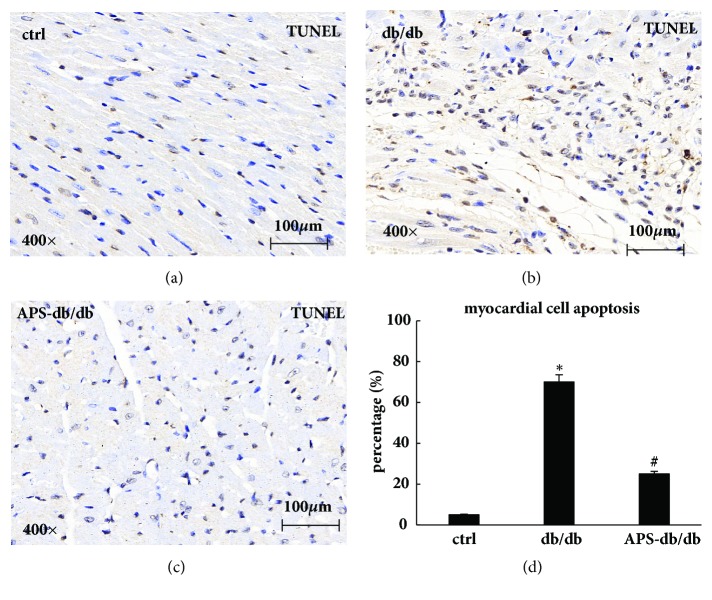
**Effects of APS on myocardial cell apoptosis in db/db mice.** Left ventricle was harvested from db/db mice with or without APS treatment (n=6 per group) after sacrifice. C57BJ/6J mice were taken as the normal control (n=6 per group). Sections of myocardium were subjected to the TUNEL assay for evaluation of apoptosis. (a)~(c) The microphotographs of TUNEL analysis in myocardium (brown stained nuclei: TUNEL staining positive, showing apoptosis; blue stained nuclei: TUNEL staining negative, showing normal). (d) The illustration for the percentage of apoptotic cardiomyocytes.

To determine ongoing apoptotic cell death, tissue sections of the left ventricular myocardium were subjected to the TUNEL assay. In contrast with the normal control hearts, more TUNEL-positive cardiomyocytes were visualized and the percentage of apoptotic cardiomyocytes increased significantly in db/db hearts (Figure 3). Overall, little nuclear DNA fragmentation by the TUNEL assay was observed in APS-treated db/db hearts, and APS treatment was able to reduce by 65% the incidence of apoptotic cardiomyocyte death in db/db mice (Figure 3), indicating a marked beneficial effect of APS on diabetes-induced myocardial cell apoptosis in diabetic mice.

**Figure 4:**
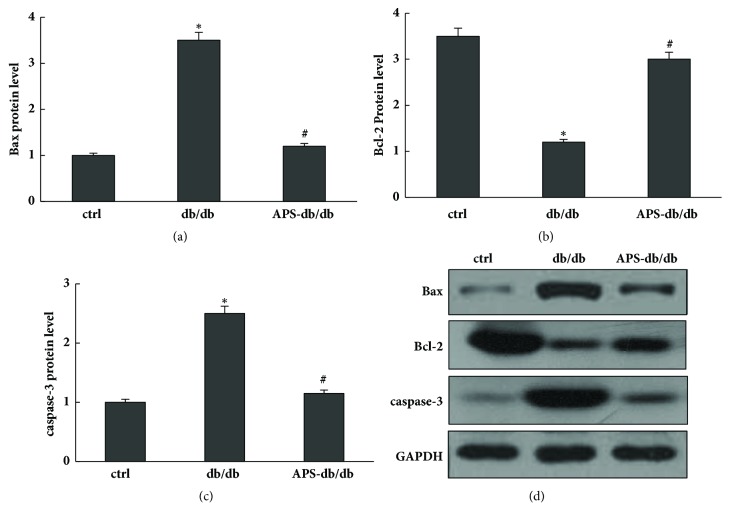
**Effects of APS on protein levels of apoptosis target genes in myocardium of db/db mice.** Left ventricular tissues obtained from C57BJ/6J mice and db/db mice with or without APS treatment (n=6 per group) were prepared and subjected to Western blotting of apoptosis target genes. Membranes were stripped and reprobed for GAPDH as a loading control. Protein content was measured respectively and normalized as a ratio relative to the normal control. (a)~(c) Protein levels of Bax, Bcl-2, and caspase-3. (d) Representative autoradiographs of Western blots. Values were presented as mean ± standard error of the mean (SEM). *∗*P<0.05 versus C57BJ/6J control mice, and #P<0.05 versus db/db mice.

Bcl-2 is a major antiapoptotic protein Bcl-2, which is thought to exert its effect at the mitochondrial outer membrane contribute to maintenance of membrane integrity. In contrast, as one of the major proapoptotic family members, Bax exerts its effects by compromising the membrane integrity leading to leakage of apoptogenic factors, resulting in caspase-3 activation which induces DNA fragmentation and apoptotic neuronal cell death. In the current study, protein expression levels of Bcl-2, Bax, and caspase-3 were evaluated respectively [22]. Our data showed that, in comparison to those of normal control, cardiac protein expression levels of Bax and caspase-3 were significantly increased in db/db mice and significantly decreased in APS-treated db/db mice, whereas that of Bcl-2 was significantly increased in db/db mice and significantly decreased in APS-treated db/db mice (Figure 4). Our results showed that APS treatment might enhance antiapoptotic protein Bcl-2 expression and inhibit apoptotic protein expression of caspase-3 and Bax in diabetic hearts.

### 3.4. Effects of APS on Cardiac ROS Formation in db/db Mice

**Figure 5:**
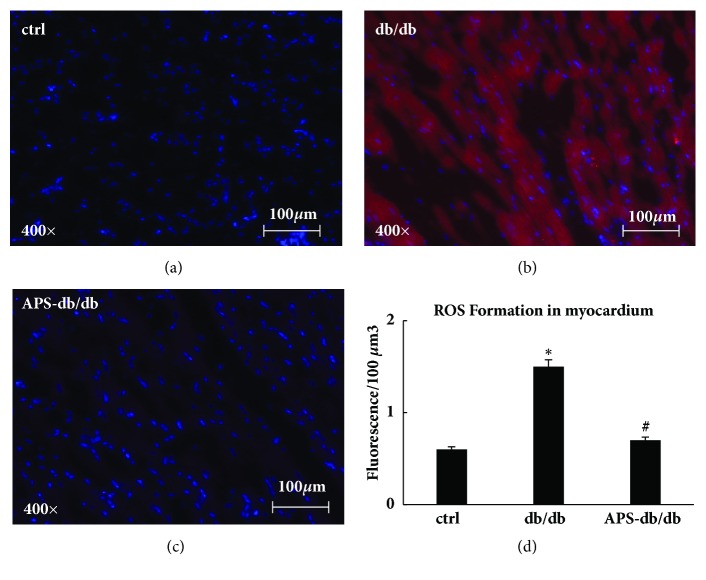
**APS inhibited ROS formation in myocardium of db/db mice.** Tissue sections of the left ventricle myocardium were obtained from C57BJ/6J mice and db/db mice with or without APS treatment (n=6 per group). CM-H_2_DCFDA was employed to measure the ROS formation, and the myocardial H_2_O_2_ and** ·**OH concentration were analyzed utilizing InSpeak Microscopy Image Intensity Calibration Microspheres and ImagePro analysis software. (a)~(c) The fluorescence microphotographs showing ROS formation in myocardium (red: fluorescence for H2O2 and** ·**OH; blue: cardiomyocytes). (d) ROS formation in myocardium from left ventricle. Values were presented as mean ± standard error of the mean (SEM). *∗*P<0.05 versus C57BJ/6J control mice, and #P<0.05 versus db/db mice.

The abundance of hydrogen peroxide (H_2_O_2_) and hydroxyl radicals (**·**OH) was measured to evaluate the ROS generation of myocardium, which were loaded with CM-H_2_DCFDA. Compared with that in the normal control, the generation of H_2_O_2_ and** ·**OH was increased by nearly 3 folds of myocardium in db/db mice (Figure 5). In contrast, cardiac ROS levels in APS-treated db/db mice were comparable with those in the normal control (Figure 5). These data showed that the cardiac ROS formation was mainly inhibited by APS treatment in db/db mice, and diabetic hearts could be effectively protected from oxidant stress by APS treatment.

### 3.5. Effects of APS on Cardiac Protein Expressions of Antioxidant Enzymes in db/db Mice

**Figure 6:**
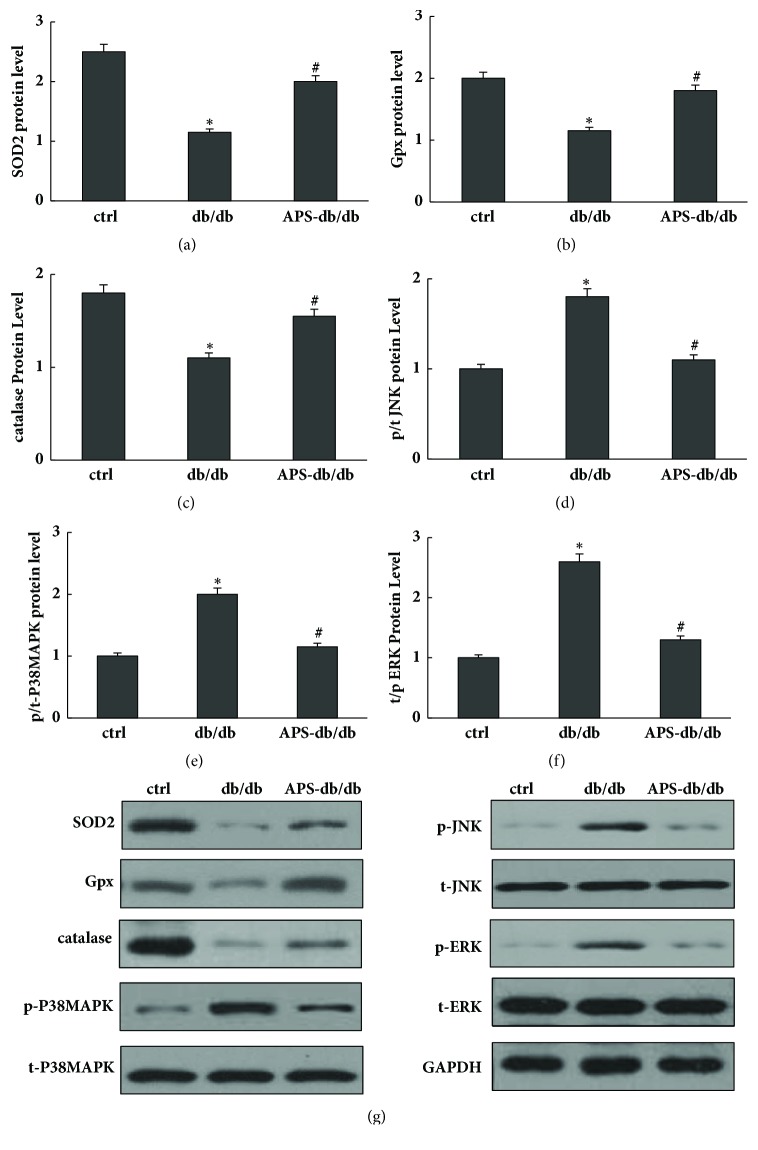
**Effects of APS on protein levels of oxidation target genes in myocardium of db/db mice.** Left ventricular tissues obtained from C57BJ/6J mice and db/db mice with or without APS treatment (n=6 samples per group) were prepared and subjected to Western blotting of oxidation target genes. Membranes were stripped and reprobed for GAPDH as a loading control. Protein content was measured respectively and normalized as a ratio relative to the normal control. (a)~(f) Protein levels of Gpx, SOD2, t-JNK, p-JNK, catalase, p-p38 MAPK, t-p38 MAPK, t-ERK, and p-ERK. (g) Representative autoradiographs of Western blots. Values were presented as mean ± standard error of the mean (SEM). *∗*P<0.05 versus C57BJ/6J control mice, and #P<0.05 versus db/db mice. Gpx: Glutathione Peroxidase; t/p-JNK: total/phosphorylated c-Jun N-terminal kinase; t/p-p38 MAPK: total/phosphorylated p38 Mitogen-activated protein kinase; t/p-ERK: total/phosphorylate extracellular signal-regulated kinase.

The magnitude of cellular oxidant stress results from a delicate balance of ROS generation and antioxidant capacity. The major form of ROS includes superoxide and hydrogen peroxide. Superoxide is converted into hydrogen peroxide by mitochondria SOD (SOD2), and hydrogen peroxide is converted to oxygen and water by catalase or glutathione peroxidase (Gpx). Western blotting assay was employed to evaluate protein expressions of major antioxidant enzymes including SOD2, catalase, and Gpx in myocardium, respectively. As shown in Figure 6, significantly lower values of protein expressions including SOD2, catalase, and Gpx were detected in db/db mice compared with those in the normal control. However, this reduction of protein levels of SOD2, catalase, and Gpx was significantly counteracted by APS treatment in db/db mice (Figure 6), suggesting the positive effects of APS on the major antioxidant system including SOD2, catalase, and Gpx in diabetic hearts.

### 3.6. Effects of APS on Cardiac Protein Expressions of MAPKs in db/db Mice

Mitogen-activated protein kinases (MAPK) are major targets of ROS, and increased oxidant stress and alteration of MAPK mainly contribute to the development of heart failure. MAPK signaling cascades are usually divided into three parallel pathways, including ERK, JNK, and p38 MAPK pathways. The protein levels of total and phosphorylated ERK, JNK, and p38 MAPK were determined by Western blotting analysis accordingly. As shown in Figure 6, in contrast to those in normal control, significant increases of cardiac p-p38 MAPK, p-ERK, and p-JNK were observed in db/db mice, as well as the ratios of the phosphorylated to total proteins. However, these increases were significant abolished by APS treatment in db/db mice, indicating that activation of JNK, ERK, and p38 MAPK might be partly suppressed by APS in diabetic hearts.

## 4. Discussion

Astragalus which is the root of Astragalus membranaceus has been widely used for the treatment of heart failure in clinical practice of traditional Chinese medicine. Astragalus polysaccharides (APS) is the main bioactive ingredient of Astragalus, and the conclusion has been proved previously [17–21]. Research has historically shown that APS could improve diabetic cardiomyopathy, both symptoms and laboratory tests [17–21]. However, the mechanism of the protective effect is still unclear and the effect of Astragalus polysaccharides on diabetes-induced oxidant stress in the diabetic cardiomyopathy remains to be explored.

In this study, we employed diabetic db/db mice treated with APS in an attempt to explore the underlying mechanism of diabetic cardiomyopathy and the protective role of Astragalus polysaccharides on cardiac dysfunction and oxidant stress in db/db mice. Our findings are in line with our previous reports that the APS can improve the derangement of structure and function in diabetic mice heart. The db/db mice exhibited significantly marked signs of systolic and diastolic ventricular dysfunction, including a significant reduction in LVEF, associated with a dilation of both LVEDD and LVESD. APS therapy significantly abolished the negative effects of ventricular hematodynamics in diabetic cardiomyopathy. In comparison with normal control mice, all parameters (including LVEDD, LVESD, and LVEF) of the APS-treated db/db mice reached the normal control values. This may suggest the prevention of APS on left ventricular dysfunction in diabetic mice. Meanwhile, there were substantive ultra-structural abnormalities of cellular mitochondria in diabetic mice hearts. However, the ultra-structural pathological changes in diabetic hearts were mainly reversed by APS treatment, which is totally different from the exhibited severe damage (including disruption of mitochondrial cristae, tubes, and sarcomeres) in the db/db mice heart, illustrating the protective effects of APS on myocardial ultra-structure in diabetic mice.

Emerging evidence supports that increased production of ROS is regarded as one of the major causes of diabetic cardiomyopathy [22–24]. Augmented ROS generation and diabetes-associated cellular senescence lead to a shift in the pattern of cell death from apoptosis to necrosis, which promotes cardinal dysfunction and fibro-blast activation. Recent research also demonstrates that metabolic impairment, generation of ROS, and enhanced cellular oxidant stress could result in myocyte apoptosis and cellular death in diabetic hearts [24]. The resulting imbalance between cell apoptosis and cell replacement favors the onset of diabetic cardiomyopathy and its progression toward heart failure. Other studies show that sustained hyperglycemia is an independent risk factor which could induce imbalance of oxidant status in cardinal cells [25]. The oxidant stress injury associated with hyperglycemia could possibly play an important pathophysiological role in diabetic cardiomyopathy. Tissue oxidant/antioxidant imbalance includes the abnormal expression of oxidant/antioxidant enzymes and the excessive activation of oxidant stress signaling system, a matter of which is a well-known implication of increased ROS generation or impaired antioxidant defenses in the heart [26]. The balance between ROS production and elimination plays a key role in preserving cardiac function. Once the delicate system is broken, the excessive myocardial ROS would impair both the myocardial function and the cardiac structure [27]. Furthermore, ROS overproduction is a trigger for myocardial apoptosis which is followed by excessive activation of apoptosis-related signaling pathways [28]. The excessive activation of apoptosis-related pathways would lead to irreversible myocardial cell damage, myocardial cell differentiation failure, and excessive apoptosis which eventually leads to diabetic myocardial remodeling and cardiac dysfunction [26–28]. The results of our previous studies showed the APS can protect both the cardiac structure and function in diabetic mice [17–21]. The underlying mechanism is still unclear, so we proposed a hypothesis in this study that the mechanism of the protective effect of APS on diabetic hearts may partly be associated with the inhibited oxidation.

Concern with this issue, our research focused on the effects of APS on diabetes-induced myocardial apoptosis in db/db mice. Our results showed that APS treatment might enhance antiapoptotic protein Bcl-2 expression and inhibit apoptotic protein expression of caspase-3 and Bax in diabetic hearts. It also showed that APS can inhibit diabetes-induced myocardial apoptosis, indicating is its protective effect on db/db diabetic hearts. Furthermore, we investigated the effects of APS on cardiac ROS formation which is an essential process of the oxidant stress injury in db/db mice. As shown by our results, the cardiac ROS formation was mainly inhibited by APS treatment in db/db mice, and diabetic hearts could be effectively protected from oxidant stress by APS treatment. It suggested that APS could remarkable prevent or at least delay the development of diabetic cardiomyopathy in db/db mice associated with inhibiting mitochondria ROS overproduction. In addition, APS may exert its effects by regulating the cardiac protein expression of antioxidant enzymes and MAPKs and thus cause the inhibition of ROS formation. Significantly lower values of protein expressions including SOD2, catalase, and Gpx were detected in db/db mice compared with those in the normal control. However, this reduction of protein levels of SOD2, catalase, and Gpx was significantly counteracted by APS treatment in db/db mice, suggesting the positive effects of APS on the major antioxidant system including SOD2, catalase, and Gpx in diabetic hearts. Moreover, we found significant increases of cardiac p-p38 MAPK, p-ERK, and p-JNK in db/db mice, as well as the ratios of the phosphorylated to total proteins. However, these increases were significantly abolished by APS treatment in db/db mice, indicating that activation of JNK, ERK, and p38 MAPK might be partly suppressed by APS in diabetic hearts.

In summary, our findings suggest that APS has benefits in diabetic cardiomyopathy, which may be partly associated with its impact on cardiac oxidant stress.

## Figures and Tables

**Table 1:** Body weight in mice.

	ctrl	db/db	APS-db/db
5 weeks old	16.28±2.04 (g)	17.03±1.64 (g)	16.62±3.22 (g)
20 weeks old	25.69±2.26 (g)	38.83±3.23 (g)*∗*	36.04±2.18 (g)*∗*

Values are mean±SEM. n=6 each group.

*∗*P<0.05 versus C57BJ/6J control mice.

There was no significant difference between db/db group and APS-db/db group.

## Data Availability

The data used to support the findings of this study are included within the article.
